# Fulminant Myocarditis With Concomitant Mycobacterium tuberculosis Presenting as a STEMI

**DOI:** 10.1016/j.jaccas.2026.107730

**Published:** 2026-04-01

**Authors:** Perneet Powar, Ahmad Gill, Shirin Jimenez

**Affiliations:** Division of Cardiology, Department of Medicine, UC Davis, Sacramento, California, USA

**Keywords:** acute coronary syndrome, acute heart failure, cardiac assist devices, cardiomyopathy, coronary angiography, echocardiography, electrocardiogram, systolic heart failure

## Abstract

**Background:**

Fulminant myocarditis is a critical inflammatory cardiomyopathy that may resemble acute coronary syndrome.

**Case Summary:**

A 76-year-old Filipino man presented with acute chest pain, with initial electrocardiogram showing inferolateral ST-segment elevations and a right bundle branch block. Coronary angiography revealed no acute culprit lesion. He developed refractory cardiogenic shock requiring mechanical circulatory support. Endomyocardial biopsy confirmed lymphocytic myocarditis, while bronchoalveolar lavage showed *Mycobacterium tuberculosis* infection. Despite treatment for both conditions, he developed multiorgan failure and died. Laboratory results were suggestive of hemophagocytic lymphohistiocytosis.

**Discussion:**

This is a rare case of fulminant myocarditis initially presenting as an ST-segment elevation myocardial infarction with no culprit vessel identified on angiography, highlighting the importance of early endomyocardial biopsy in cases of unexplained cardiogenic shock.

**Take-Home Messages:**

Fulminant myocarditis is a rare cause of cardiogenic shock, while hemophagocytic lymphohistiocytosis is a rare complication of tuberculosis. Multidisciplinary care is critical to diagnosing and treating these uncommon but morbid conditions.

## Visual Summary

**Figure:**
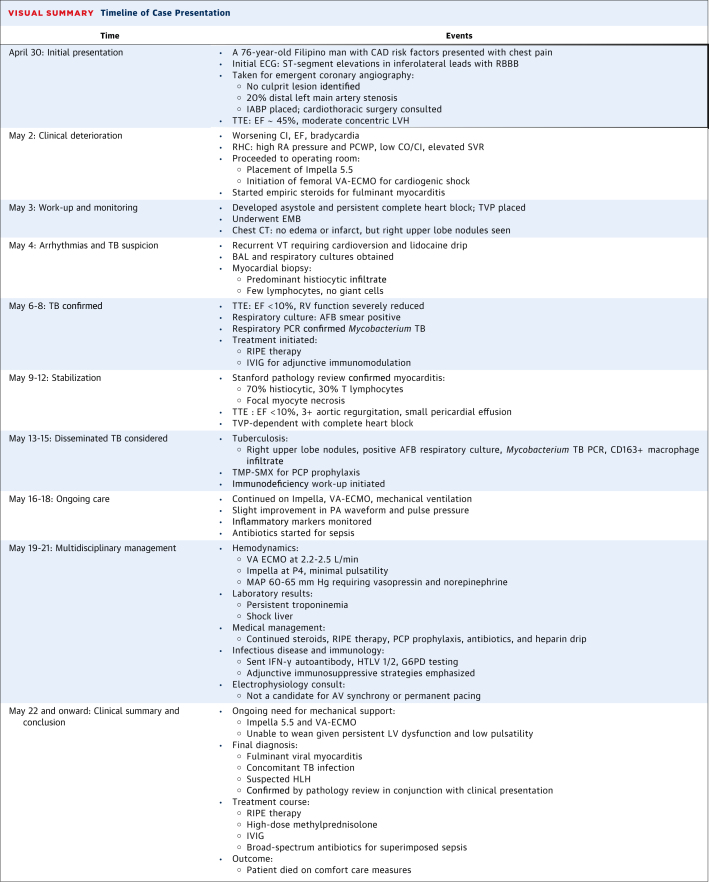

## History of Presentation

**Figure 1:**
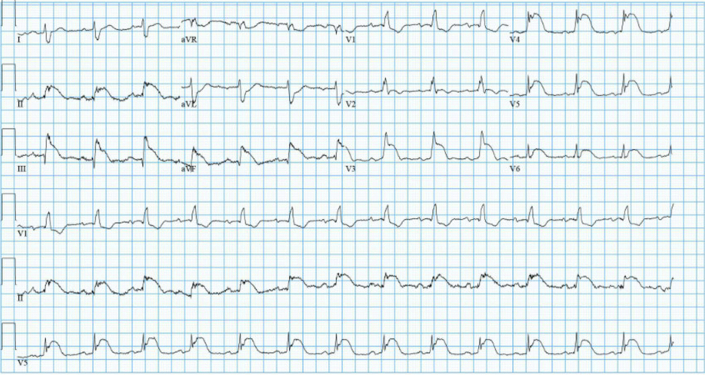
Initial Electrocardiogram Initial electrocardiogram displayed ST-segment elevations in leads V_3_ to V_6_ and II, III, aVF, with a right bundle branch block.

**Figure 2:**
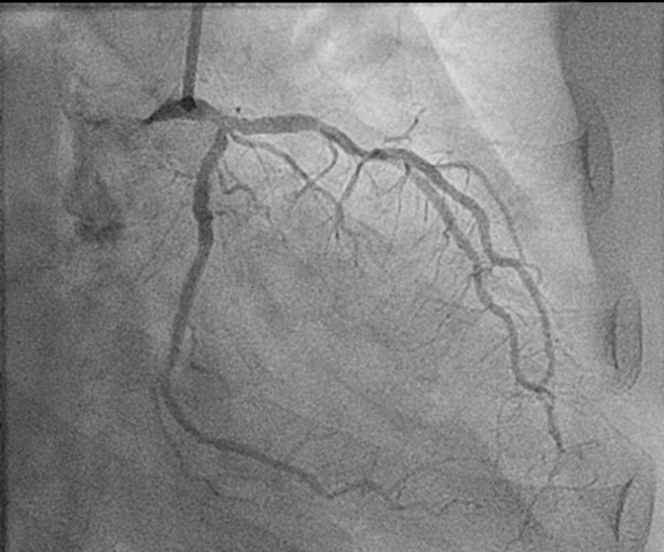
Initial Cardiac Catheterization Showing the Left Coronary System Findings included 20% stenosis of the distal left main coronary artery and 40% stenosis of the first diagonal branch of the left anterior descending and proximal circumflex arteries.

**Figure 3:**
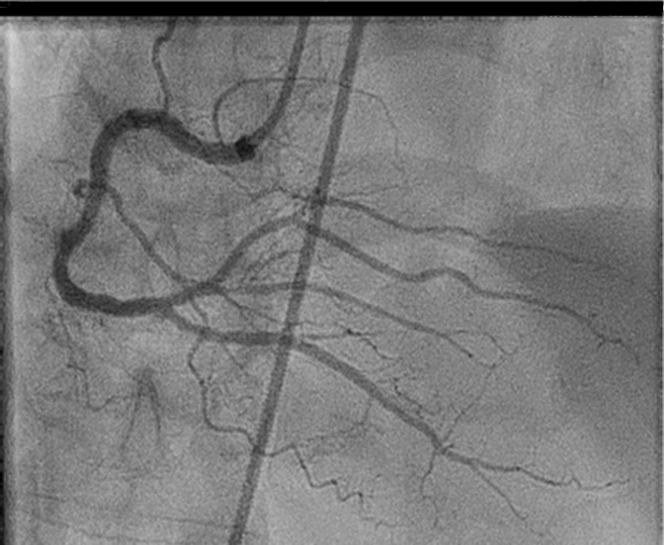
Initial Cardiac Catheterization Showing the Right Coronary System Findings included 40% stenosis of the proximal right coronary artery.

A 76-year-old Filipino man presented to the emergency department endorsing acute cardiac chest pain for 3 days, with an unremarkable physical examination. Initial electrocardiogram showed inferolateral ST-segment elevation myocardial infarction (STEMI) with a new right bundle branch block (Figure 1). Cardiac catheterization revealed 20% stenosis in the distal left main coronary artery and 40% stenosis in the first diagonal branch of the left anterior descending, proximal left circumflex, and proximal right coronary arteries, without a culprit lesion (Figures 2 and 3).

**Take-Home Messages**

• Fulminant myocarditis is a rare cause of cardiogenic shock, while hemophagocytic lymphohistiocytosis is a rare complication of tuberculosis.
• Multidisciplinary care is critical to diagnosing and treating these uncommon but morbid conditions.

**Figure 4:**
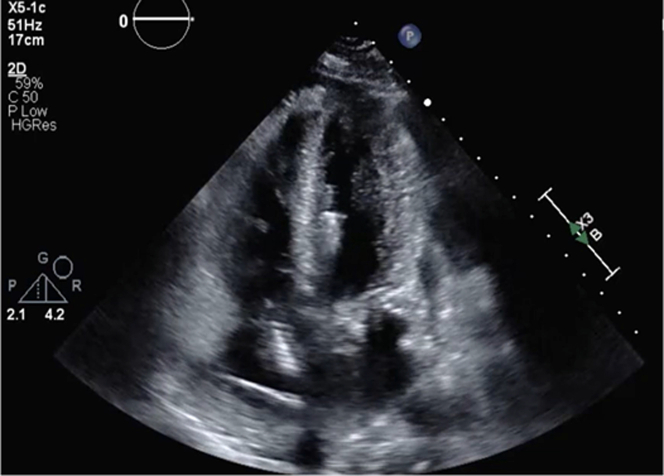
Initial Transthoracic Echocardiogram Findings included an ejection fraction of 45%, moderate concentric left ventricular hypertrophy, and hypokinetic apical inferior septum/anterior segments.

**Figure 5:**
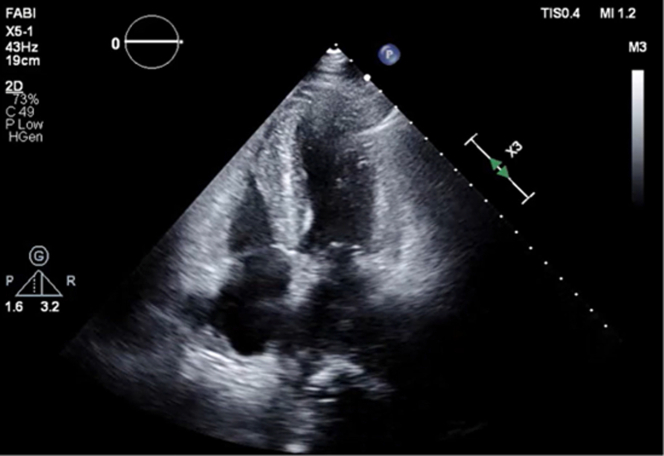
Repeat Transthoracic Echocardiogram Findings included severely reduced left ventricular ejection fraction of 25% and biventricular failure, small pericardial effusion, pacing wires, and left ventricular assist device.

Initial hemodynamics via right heart catheterization (RHC) revealed elevated right- and left-sided pressures, low cardiac output/index, and high systemic vascular resistance (right atrial pressure: 17 mm Hg, right ventricular pressure: 35/18 mm Hg, pulmonary artery pressure: 36/21/28 mm Hg, pulmonary capillary wedge pressure: 22 mm Hg, cardiac output: 3.6 L/min, cardiac index: 2.11 L/min/m^2^). Given the concern for shock and left main disease, an intra-aortic balloon pump (IABP) was placed. Transthoracic echocardiography (TTE) showed an ejection fraction of 45%, with mildly reduced right ventricular systolic function (Figure 4). The patient was evaluated by cardiothoracic surgery, with initial plans to undergo coronary artery bypass grafting. However, within 48 hours, he developed rapidly progressive cardiogenic shock with rising cardiac biomarkers, persistent ST-segment elevations on electrocardiogram out of proportion to his coronary artery disease, and new bradycardia. He underwent repeat RHC, which revealed severe cardiogenic shock (right arterial pressure: 15 mm Hg, right ventricular pressure: 32/19 mm Hg, pulmonary artery pressure: 33/26/18 mm Hg, pulmonary capillary wedge pressure: 17 mm Hg, cardiac index:1.3 L/min/m^2^, and high systemic vascular resistance: 2,280 dyn⋅s⋅cm^–5^). Repeat TTE showed worsening biventricular heart failure with an ejection fraction of 25% to 30% with septal and apical hypokinesis, requiring an upgrade from his IABP to an Impella 5.5 (Abiomed) (Figure 5).

During Impella placement, the patient developed sustained ventricular tachycardia requiring direct current cardioversion. He then became hypotensive and was initiated on femoral venoarterial extracorporeal membrane oxygenation. High-dose steroids were started for empiric treatment of suspected fulminant myocarditis. Later that night, he was noted to be in complete heart block with a native heart rate in the 20s to 30s, followed by a period of asystole.

## Past Medical History

The patient had a history of hypertension, type 2 diabetes mellitus, and right frontal cavernomas.

## Differential Diagnosis

Considerations included STEMI; myocarditis, including giant cell, lymphocytic/tuberculosis (TB), viral, drug-induced, and hemophagocytic lymphohistiocytosis (HLH); fulminant myocarditis; and pericarditis.

## Investigations

**Figure 6:**
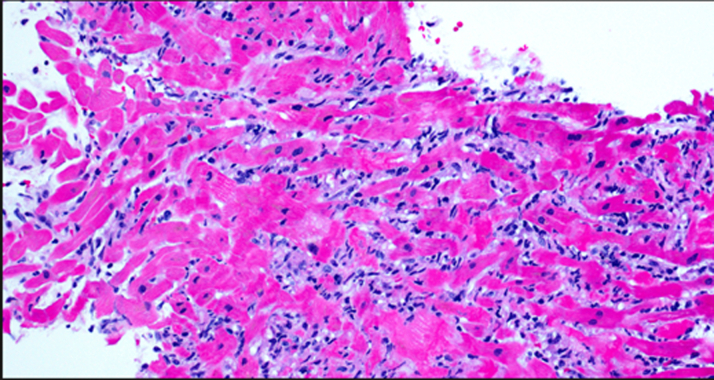
Pathology From Endomyocardial Biopsy Results showed heavy histiocytic (70%) and T lymphocytic (30%) interstitial infiltrate, consistent with myocardial necrosis. CD163 and CD3 immunohistochemical stains were consistent with severe lymphocytic myocarditis.

After complete heart block, the patient was returned to the cardiac catheterization laboratory for transvenous pacing and endomyocardial biopsy (EMB) (Figure 6). The EMB showed a heavy histiocytic and T lymphocytic interstitial infiltrate with focal myocyte necrosis, suggestive of viral myocarditis. Further pathology review confirmed myocyte necrosis and CD163+ macrophage infiltrate, while myocardial tissue polymerase chain reaction (PCR) was negative for *Mycobacterium tuberculosis* bacteria. Chest computed tomography and bronchoscopy findings of the right upper lobe lung nodules with positive acid-fast bacillus testing via bronchoalveolar lavage confirmed a diagnosis of *Mycobacterium* TB infection. Immunology laboratory tests were significant for CXCL9 of 19,421 (high) and IL-2 (CD25) marker of 1894 (high), which in combination with cytopenias, fever, and abnormal liver tests suggested a diagnosis of HLH. The patient was too unstable for a bone marrow biopsy to be completed, thus he did not meet the full diagnostic criteria for HLH via H-score or HLH-2004 criteria assessment. Supportive laboratory findings such as ferritin, triglycerides, and fibrinogen were normal, although they were collected after starting steroids and may have been falsely negative.

## Management

The patient was initially referred to cardiothoracic surgery for consideration of coronary artery bypass grafting based on cardiac catheterization findings. He required mechanical circulatory support with IABP, Impella, and subsequently extracorporeal membrane oxygenation given clinical progression of severe cardiomyopathy. He received steroids for suspected fulminant myocarditis. Despite empiric treatment, he required transvenous pacing for asystole and cardioversion with lidocaine drip for sustained ventricular tachycardia. After respiratory cultures and PCR confirmed *Mycobacterium* TB, he was started on RIPE (rifampin, isoniazid, pyrazinamide, ethambutol) therapy and vitamin B6 for disseminated TB in addition to high-dose steroids and intravenous immunoglobulin for immunomodulation. He was also started on trimethoprim-sulfamethoxazole for *Pneumocystis* pneumonia prophylaxis. Ultimately, he required vasopressors, broad-spectrum antibiotics, and supportive transfusions for mixed shock and TB infiltration.

## Outcome and Follow-Up

Despite appropriate therapy, the patient continued to deteriorate clinically, developing sepsis and having further episodes of sustained ventricular tachycardia. He was unable to be weaned off mechanical support and vasopressors. Ultimately, the patient was transitioned to comfort care measures and died on hospital day 21 from fulminant myocarditis.

## Discussion

This case represents an antemortem diagnosis of fulminant myocarditis that initially presented as a STEMI. ST-segment elevation in myocarditis often reflects epicardial inflammation, yet it is indistinguishable from acute coronary syndrome without angiography.^1^ The absence of obstructive coronary artery disease should prompt early consideration of alternative diagnoses, particularly in patients with rapid hemodynamic deterioration.^2^

This patient had lymphocytic myocarditis leading to reactivation of TB in the setting of critical illness and steroid treatment. This likely led him to develop HLH, which is a rare but highly morbid complication of disseminated TB. Histologically, TB typically demonstrates caseating granulomas; however, this patient's myocardial biopsy revealed a predominantly histiocytic infiltrate comprising 70% CD163+ macrophages and 30% T-cells, with focal myocyte necrosis more consistent with a viral etiology.^3^ Positive immune testing with CXCL9 and IL2 in addition to clinical findings of cytopenia, fever, and hepatic dysfunction led to suspicion of HLH in the setting of disseminated TB, with fulminant myocarditis occurring via immune-mediated response. The classification of sporadic versus familial HLH is important, as treatment of underlying conditions can be sufficient for full recovery. TB is implicated in 9% to 25% of HLH cases, making it rare but potentially fatal.

While cases can be responsive to treatment, it does not reduce the risk of sudden cardiac death, and a large majority of cases are diagnosed postmortem given the high lethality of fulminant myocarditis and the delay in diagnosing HLH.^4^ EMB in addition to TB PCR should be completed promptly to improve detection. Early diagnosis of HLH is difficult because of nonspecific presenting signs and symptoms. Timely bone marrow biopsy may be beneficial in cases of diagnostic uncertainty. Conduction abnormalities and arrhythmias are associated with high mortality in fulminant myocarditis.^5^ Corticosteroids remain standard treatment for many manifestations of TB, HLH, and severe myocarditis, however in the present case the treatment with high-dose steroids led to immunosuppression that likely contributed to reactivation of latent TB.

In addition to anti-TB drug therapy, symptomatic relief of cardiac abnormalities with mechanical ventilation, inotropic agents, and vasopressors should be undertaken. Future research should evaluate whether earlier TB-directed therapy, cytokine-targeted immunomodulation, or earlier biventricular support could improve outcomes in this devastating condition.^6^^,^^7^

## Conclusions

This study presents a rare case of fulminant myocarditis with concomitant *Mycobacterium* TB and HLH presenting as a STEMI. Viral myocarditis can be a lethal cause of fulminant myocarditis that can mimic acute coronary syndrome. Our findings highlight the need for maintaining a high index of suspicion in STEMI presentations without obstructive coronary artery disease, especially in patients with risk factors for consequential infections such as TB. Early EMB with TB testing in patients with risk factors can facilitate timely diagnosis and treatment.

## Funding Support and Author Disclosures

The authors have reported that they have no relationships relevant to the contents of this paper to disclose.

**Visual Summary:** Timeline of Case Presentation

Time	Events
April 30: Initial presentation	• A 76-year-old Filipino man with CAD risk factors presented with chest pain • Initial ECG: ST-segment elevations in inferolateral leads with RBBB • Taken for emergent coronary angiography: ○ No culprit lesion identified ○ 20% distal left main artery stenosis ○ IABP placed; cardiothoracic surgery consulted • TTE: EF ∼45%, moderate concentric LVH
May 2: Clinical deterioration	• Worsening CI, EF, bradycardia • RHC: high RA pressure and PCWP, low CO/CI, elevated SVR • Proceeded to operating room: ○ Placement of Impella 5.5 ○ Initiation of femoral VA-ECMO for cardiogenic shock • Started empiric steroids for fulminant myocarditis
May 3: Work-up and monitoring	• Developed asystole and persistent complete heart block; TVP placed • Underwent EMB • Chest CT: no edema or infarct, but right upper lobe nodules seen
May 4: Arrhythmias and TB suspicion	• Recurrent VT requiring cardioversion and lidocaine drip • BAL and respiratory cultures obtained • Myocardial biopsy: ○ Predominant histiocytic infiltrate ○ Few lymphocytes, no giant cells
May 6-8: TB confirmed	• TTE: EF <10%, RV function severely reduced • Respiratory culture: AFB smear positive • Respiratory PCR confirmed *Mycobacterium* TB • Treatment initiated: ○ RIPE therapy ○ IVIG for adjunctive immunomodulation
May 9-12: Stabilization	• Stanford pathology review confirmed myocarditis: ○ 70% histiocytic, 30% T lymphocytes ○ Focal myocyte necrosis • TTE: EF <10%, 3+ aortic regurgitation, small pericardial effusion • TVP-dependent with complete heart block
May 13-15: Disseminated TB considered	• Tuberculosis: ○ Right upper lobe nodules, positive AFB respiratory culture, *Mycobacterium* TB PCR, CD163+ macrophage infiltrate • TMP-SMX for PCP prophylaxis • Immunodeficiency work-up initiated
May 16-18: Ongoing care	• Continued on Impella, VA-ECMO, mechanical ventilation • Slight improvement in PA waveform and pulse pressure • Inflammatory markers monitored • Antibiotics started for sepsis
May 19-21: Multidisciplinary management	• Hemodynamics: ○ VA ECMO at 2.2-2.5 L/min ○ Impella at P4, minimal pulsatility ○ MAP 60-65 mm Hg requiring vasopressin and norepinephrine • Laboratory results: ○ Persistent troponinemia ○ Shock liver • Medical management: ○ Continued steroids, RIPE therapy, PCP prophylaxis, antibiotics, and heparin drip • Infectious disease and immunology: ○ Sent IFN-γ autoantibody, HTLV 1/2, G6PD testing ○ Adjunctive immunosuppressive strategies emphasized • Electrophysiology consult: ○ Not a candidate for AV synchrony or permanent pacing
May 22 and onward: Clinical summary and conclusion	• Ongoing need for mechanical support: ○ Impella 5.5 and VA-ECMO ○ Unable to wean given persistent LV dysfunction and low pulsatility • Final diagnosis: ○ Fulminant viral myocarditis ○ Concomitant TB infection ○ Suspected HLH ○ Confirmed by pathology review in conjunction with clinical presentation • Treatment course: ○ RIPE therapy ○ High-dose methylprednisolone ○ IVIG ○ Broad-spectrum antibiotics for superimposed sepsis • Outcome: ○ Patient died on comfort care measures

AFB = acid-fast bacilli; AV = atrioventricular; BAL = bronchoalveolar lavage; CAD = coronary artery disease; CI = cardiac index; CO = cardiac output; CT = computed tomography; ECG = electrocardiogram; EF = ejection fraction; EMB = endomyocardial biopsy; HLH = hemophagocytic lymphohistiocytosis; IABP = intra-aortic balloon pump; IVIG = intravenous immunoglobulin; LV = left ventricular; LVH = left ventricular hypertrophy; MAP = mean arterial pressure; PA = pulmonary artery; PCP = *Pneumocystis* pneumonia; PCR = polymerase chain reaction; PCWP = pulmonary capillary wedge pressure; RA = right atrial; RBBB = right bundle branch block; RHC = right heart catheterization; RIPE = rifampin, isoniazid, pyrazinamide, ethambutol; RV = right ventricular; SVR = systemic vascular resistance; TB = tuberculosis; TMP-SMX = trimethoprim-sulfamethoxazole; TTE = transthoracic echocardiography; TVP = transvenous pacing; VA-ECMO = venoarterial extracorporeal membrane oxygenation; VT = ventricular tachycardia.

## References

[1] Ammirati E., Frigerio M., Adler E.D. (2020). Management of acute myocarditis and chronic inflammatory cardiomyopathy: an expert consensus document. Circ Heart Fail.

[2] Kociol R.D., Cooper L.T., Fang J.C. (2020). Recognition and initial management of fulminant Myocarditis. Circulation.

[3] Cowley A., Dobson L., Kurian J. (2017). Acute myocarditis secondary to cardiac tuberculosis: a case report. Echo Res Pract.

[4] McCarthy R.E., Boehmer J.P., Hruban R.H. (2000). Long-term outcome of fulminant myocarditis. N Engl J Med.

[5] Tschöpe C., Ammirati E., Bozkurt B. (2021). Myocarditis and inflammatory cardiomyopathy. Nat Rev Cardiol.

[6] Toldo S., Abbate A. (2018). The NLRP3 inflammasome in acute MI. Nat Rev Cardiol.

[7] Combes A., Price S., Slutsky A.S., Brodie D. (2020). Temporary circulatory support for cardiogenic shock. Lancet.

